# Herbal Medicine Usage During the COVID-19 Pandemic in Indonesia: Trends and Determinants

**DOI:** 10.1155/tswj/1639500

**Published:** 2025-05-14

**Authors:** Erna Harfiani, Ratna Puspita, Isniani Ramadhani Sekar Prabarini

**Affiliations:** ^1^Department of Pharmacy and Pharmacology, Faculty of Medicine, UPN Veteran Jakarta, Jakarta, Indonesia; ^2^Department of Biochemistry, Faculty of Medicine, UPN Veteran Jakarta, Jakarta, Indonesia; ^3^Program of Ethics Medicolegal, Faculty of Medicine, UPN Veteran Jakarta, Jakarta, Indonesia

**Keywords:** alternative medicine, COVID-19, herbal, pandemic, preventive medicine

## Abstract

**Background:** The COVID-19 pandemic led to increased use of herbal medicine in Indonesia, driven by its perceived efficacy in enhancing immunity. This study examines the sociodemographic factors influencing herbal medicine consumption during the pandemic.

**Methods:** A cross-sectional survey was conducted from June to July 2021 across 33 Indonesian provinces, involving 461 respondents. Key sociodemographic variables included gender, age, education, domicile, and occupation, while the dependent variable was herbal medicine consumption. Data were analyzed using univariate and bivariate analyses, with significance determined by *p* values.

**Results:** A total of 62.7% of respondents reported using herbal medicine during the COVID-19 pandemic, followed by standardized herbal medicine (23.2%) and phytopharmaceuticals (14.1%). Among the respondents, 66.4% were female, and 29.9% were aged 17–25. Herbal medicine consumption was significantly influenced by age (*p* = 0.006) and occupation (*p* = 0.038). Students (26.2%) and individuals on Java Island (62.7%) constituted the largest consumer groups. Key ingredients included ginger, turmeric, and lime, which are widely recognized for their antiviral and immunomodulatory properties.

**Conclusion:** Age and occupation were identified as associated factors of herbal medicine use, emphasizing the importance of tailored public health strategies to promote traditional remedies as complementary measures. These findings highlight herbal medicine's cultural and therapeutic relevance during health crises.

## 1. Introduction

SARS-CoV-2 is a virus that causes respiratory illness in humans. It uses the same receptor, Angiotensin-Converting Enzyme 2 (ACE2), as SARS-CoV to infect human respiratory cells [1, 2]. The virus is primarily transmitted through respiratory droplets and aerosols but can also be transmitted through direct contact with contaminated surfaces and fecal–oral transmission [2]. In severe cases, SARS-CoV-2 can trigger an immune response that leads to inflammation and the release of proinflammatory cytokines, which can have various consequences such as multiorgan dysfunction, acute respiratory distress syndrome (ARDS), and cytokine storms [3, 4]. The virus can infect the upper and lower respiratory tract cells, leading to inflammation and limiting gas exchange [3, 4]. The virus can infect pulmonary capillary endothelial cells, compromising the integrity of the epithelial–endothelial barrier [1]. There is no definite, consolidated, and efficient cure for COVID-19 infection. Several types of vaccines are currently available to inhibit the COVID-19 pandemic, but their delivery is still challenging, especially in developing countries [5]. Therefore, the researchers' primary concern is developing reliable and effective antiviral therapy for SARS-CoV-2. Jamu can be an ideal gateway to find effective anti-COVID-19 therapies [6]. We have explicitly articulated the research gap by stating that while herbal medicine has been widely recognized globally, there is limited understanding of how sociodemographic factors influence its consumption during health crises, particularly in Indonesia. We have compared Indonesia's reliance on herbal medicine with other countries, noting that many nations, especially in Asia and Africa, have seen a resurgence in traditional remedies during the COVID-19 pandemic due to their accessibility and cultural familiarity.

The use of herbal medicine has been influenced by various factors, as highlighted in the existing literature. Previous studies have identified factors such as cultural beliefs, accessibility, perceived efficacy, and the influence of family and community as significant determinants of herbal medicine use. Understanding these factors is crucial, as they provide insight into the motivations behind individuals' choices to utilize herbal remedies. Furthermore, examining sociodemographic factors—such as age, gender, occupation, and education level—is essential in our study. These factors can significantly impact health-seeking behaviours and preferences for herbal medicine, allowing for a more comprehensive understanding of the population's reliance on traditional remedies [7]. By integrating these elements into our research, we aim to provide a nuanced perspective on the dynamics of herbal medicine use during the COVID-19 pandemic in Indonesia.

People in many countries, especially in Asia and Africa, have used medicinal plants for centuries to treat various ailments due to their availability and relatively low cost. In China and other Asian countries, traditional medicine has been widely used and studied. In Africa, approximately 60%–80% of the population relies on traditional remedies to treat themselves for various diseases, with herbal therapy being the most common form of traditional medicine used in sub-Saharan Africa [8–10]. Thus, developing new drugs with possible anti-COVID-19 efficacy from herbs and their bioactive components is possible [11]. We emphasized that Jamu is a traditional herbal concoction based on empirical knowledge, while standard herbal medicine (OHT) and phytopharmaceuticals are more standardized and scientifically validated.

Phytochemical metabolites such as tannins, terpenoids, alkaloids, coumarins, flavonoids, and polyphenols have shown efficacy against pathogenic microorganisms by inhibiting viral enzymatic and protein activity, thereby inhibiting viral entry and replication in affected host cells. Several reports recommend the effectiveness of herbal bioactive compounds in reducing and managing the risk of SARS-CoV-2 [5, 12–14]. Jamu, the Indonesian traditional herbal medicine, has been studied for its potential use in dealing with COVID-19. Some jamu formulas have antioxidant and anti-inflammatory effects that could be promising for COVID-19 treatment [15]. Treatment systems in various geographic zones use traditional herbal medicine as the primary treatment for viral infections, including those triggered by SARS-CoV. For example, in Indonesia, a group called TOGA (family medicinal plants) is often planted in the yard for daily life [16]. Therefore, this study aims to determine the sociodemographic characteristics of the Indonesian people that influence herbal medicine consumption during the COVID-19 pandemic so that it can be developed into food supplements and functional foods with potential antiviral activity related to COVID-19.

## 2. Methods

### 2.1. Research Design

This study utilized an analytical descriptive approach with a cross-sectional survey design conducted from June to July 2021 in 33 provinces of Indonesia. The research explored the relationship between sociodemographic factors (gender, age, education level, domicile, and occupation) and herbal medicine consumption during the COVID-19 pandemic. The dependent variable was the use of herbal medicines, categorized into three classes: traditional herbal (jamu), OHT, and phytopharmaceuticals. This study was approved by the Faculty of Medicine ethics committee, UPN Veterans Jakarta, with reference number 449/IX/2021/KEPK. Ethical considerations were paramount in this study; we ensured participant anonymity and obtained informed consent before participation. Participants were informed about the purpose of the study and their right to withdraw at any time without consequence.

### 2.2. Population and Research Sample

The target population consisted of Indonesian residents aged 17–65 who had previously consumed herbal medicines. The cluster sampling method employed in this study was designed to capture a diverse range of Indonesian demographics, ensuring representation from rural and urban populations. An analysis of the demographic data collected indicates that our sample adequately reflects the diversity of the Indonesian population. The sampling method used was cluster sampling, ensuring representation across different regions. A total of 461 respondents participated in the study. The sample size was justified based on considerations of Indonesia's large and diverse population, expected response variability, and sufficient statistical power for bivariate analyses. Inclusion criteria required participants to consume herbal medicine and provide informed consent. Respondents who had never used herbal medicine or did not complete the questionnaire were excluded.

The target population consisted of Indonesian residents aged 17–65 who had previously consumed herbal medicines. The sampling method used was cluster sampling, ensuring representation across different regions. A total of 461 respondents participated in the study. The sample size was justified based on considerations of Indonesia's large and diverse population, expected response variability, and sufficient statistical power for bivariate analyses.

The age range of 17–65 years was chosen to align with the study's objective of understanding herbal medicine consumption patterns among adults capable of making independent health decisions. Adolescents under 17 were excluded due to reliance on parental guidance for health choices, while those above 65 were excluded as their health decisions often involved prescribed pharmaceuticals. This range ensures inclusivity of key consumer groups, such as young adults and middle-aged individuals, who are more likely to use traditional medicine for preventive health measures during the pandemic.

The inclusion criteria for this study involved individuals aged 17–65 who had prior experience consuming herbal medicine and were willing to complete the questionnaire independently. As part of the exclusion criteria, this study did not include individuals without formal basic education. This exclusion was implemented to ensure that respondents comprehended the questionnaire content and the study's objectives, thereby enhancing the relevance and validity of the collected data. Additionally, individuals who had never consumed herbal medicine were excluded to avoid data bias and to support the analysis of correlations between education, knowledge, and herbal medicine consumption.

We acknowledge the concern regarding the potential misalignment of our exclusion and inclusion criteria. To clarify, they decided to exclude adolescents under 17 due to their reliance on parental guidance for health-related decisions. Similarly, individuals over the age of 65 were excluded as their health choices often involve prescribed pharmaceuticals, which may not accurately reflect the use of herbal medicine.

Furthermore, we recognize that knowledge is a fundamental component of health practice modifications, particularly during a pandemic [17–19]. To mitigate potential bias arising from the exclusion of individuals without formal education, we will conduct a stratified analysis to examine how educational background influences health practices and attitudes towards herbal medicine use. This methodological approach aims to produce reliable data and accurately reflect demographic trends, ensuring a comprehensive understanding of the factors influencing herbal medicine consumption.

### 2.3. Research Instruments

The survey instrument consisted of a structured online questionnaire divided into two sections: (1) sociodemographic characteristics, including gender, age, education level, domicile, and occupation, and (2) herbal medicine use, focusing on the types of herbal medicines consumed, specific ingredients, and patterns of consumption. The questionnaire was designed based on prior literature and underwent a pilot test with 40 participants to validate its clarity and reliability. The instrument achieved high internal consistency scores in the pilot phase.

### 2.4. Operational Definition

Key variables were defined as follows:

• Gender: male or female.
• Age: categorized into 17–25, 26–35, 36–45, 46–55, and 56–65 years.
• Education level: elementary–high school, diploma, undergraduate (S1), and postgraduate (S2/S3).
• Domicile: geographic location categorized by major islands.
• Occupation: grouped into students, workers, entrepreneurs, homemakers, and others.
• Herbal medicine: categorized into three classes: jamu/herbal, OHT, and phytopharmaceuticals.

### 2.5. Data Collection

Data were collected through an online survey using Google Forms and distributed via WhatsApp to reach a broad demographic. Participants were required to complete an informed consent form before accessing the questionnaire. While data collection was conducted using Google Forms and WhatsApp, we recognize that this digital-only approach may introduce potential bias, as it could limit participation from individuals without reliable Internet access, particularly in rural areas. The survey link was disseminated through social media and community networks to maximize participation. Data were compiled in Microsoft Excel and processed for further analysis.

### 2.6. Data Analysis

In this study, statistical analyses were conducted using SPSS software, version 26.0 (IBM Corp., Armonk, NY, United States). This version of SPSS was utilized to perform descriptive statistics, correlation analyses, and regression analyses to evaluate the relationships between the variables of interest. The choice of this software version ensures that the analysis is conducted with the latest features and capabilities available for robust statistical evaluation. Data analysis involved two stages:

1. Univariate analysis: Descriptive statistics were used to summarize sociodemographic characteristics and patterns of herbal medicine consumption.
2. Bivariate analysis: Chi-square tests were applied to determine the relationship between sociodemographic variables and herbal medicine consumption. Statistical significance was set at *p* < 0.05. Analysis was performed using SPSS software, ensuring robust statistical evaluation.

## 3. Result

### 3.1. Characteristics

Among the 461 respondents, females (66.4%) consumed more herbal medicines than males (33.6%), and the dominant age group was 17–25 years (29.9%), followed by 26–35 years (24.5%) and 56–65 years (7.4%). Based on the regions, people living on the island of Java (62%) had a higher consumption rate compared to those in other areas (37.3%), such as the islands of Sumatra, Bali, NTB, Kalimantan, Sulawesi, and Papua. For the work variable, student respondents (26.2%) were the dominant consumer group during the COVID-19 pandemic, followed by the self-employed (22.6%), workers (22.4%), housewives (11.9%), and others (16.9%), as shown in Table 1.

### 3.2. Classification of the Use of Herbal Class Medicine

There is a classification for herbal medicines based on testing and standardization of their ingredients, namely, jamu/herbal, OHT, and phytopharmaceuticals.  Table 2 shows that the herbal/herbal was the most consumed type by 62.7% of the participants, followed by OHT and phytopharmaceuticals by 23.2% and 14.1%, respectively.

Data were also obtained on the ingredients for the various classes of medicine, where the three most widely used were ginger/red ginger, turmeric, and lime. Others include garlic, lemongrass, curcuma, *habatussauda*, moringa, betel, celery, guava, and bitter *brotowali*. Furthermore, for the herbal form class, the three most common ingredients are turmeric tamarind, ginger tea, and green tea, followed by *wedding uwuh*, *tampon tampon*, VCO, Rosella tea, cajuput candy, homemade herbal concoction, and kencur rice. In the OHT class, the top three products that are often used during the pandemic were Tolak Angin, Antangin JRG, and OBH Herbal, followed by Kiranti, Sehat segar, and other OHT, such as Herbacold, Mastin, and Dehaf. The three classes of phytopharmaceuticals that were most consumed include Stimuno, Redacid, and Tensigard, followed by Disolf, Diabetatdex, Vipalbumin, Rheumeneer, and XGra, as shown in Table 3.

The classification of herbal medicines in Table 3 into Jamu, OHT, and phytopharmaceuticals follows the Indonesian regulatory framework for traditional medicines. Jamu refers to herbal concoctions based on empirical use, prepared with simple techniques and consumed without extensive scientific validation. OHT, on the other hand, includes OHTs that have undergone preclinical testing to ensure safety and efficacy. Phytopharmaceuticals represent the most scientifically validated category, involving clinical trials and standardized ingredients to meet pharmaceutical-grade standards.

The listed herbal brands, such as Tolak Angin and Stimuno, represent popular choices in their respective categories, reflecting broader consumer preferences during the pandemic. However, the consumption patterns may be influenced by regional and socioeconomic factors. For instance, accessibility to phytopharmaceuticals may be limited in rural or economically disadvantaged areas, where Jamu remains more prevalent due to its affordability and cultural familiarity. Similarly, urban regions may consume more OHT and phytopharmaceuticals due to better market availability and health literacy. This suggests that while the listed brands capture a general trend, preferences might vary significantly based on geographical, economic, and cultural contexts. Future studies should explore these dynamics to provide a nuanced understanding of how such factors shape herbal medicine consumption patterns in Indonesia.

### 3.3. Time for Herbal Medicine Consumption

In Indonesia, people have been consuming herbal medicine for generations due to its various benefits. The occurrence of the COVID-19 pandemic led to an increase in the rate of consumption. The results showed that 199 respondents (43.2) often used herbal medicine before the pandemic. Furthermore, there was an increase in the number of consumers to 262 (56.8) after the pandemic started in late 2019 to early 2020, where 15.4 and 41.4 of them consumed the medicine for 2–3 years and 1 year, respectively, as shown in Table 4.

### 3.4. The Relationship Between Sociodemographic Characteristics and the Herbal Medicine Consumption

The results showed that 66.4% of participants were females aged 17–25 (29.9) and undergraduates (40.1) with *p* values of 0.05, 0.006, and 0.005, respectively. Based on the domicile factor, 62.7% of people who use herbal medicines lived on the island of Java (*p* value 0.700), followed by the islands of Sumatra, Sulawesi, Papua, Kalimantan, Bali, and NTB. Meanwhile, for occupational factors, 26.2% were students (*p* value 0.038), followed by workers and entrepreneurs. The most widely used class of medicine was the herbal form, as shown in Table 5.

## 4. Discussion

### 4.1. The Herbal Medicine Consumption in Indonesian Society

The study uniquely highlights the sociodemographic determinants influencing herbal medicine consumption during the COVID-19 pandemic in Indonesia, a topic scarcely explored at this scale. By using a cross-sectional survey across 33 Indonesian provinces, it identifies age and occupation as key factors significantly associated with herbal consumption. The study's findings align with global trends in alternative medicine use during crises, such as increased reliance on traditional remedies in Asia and Africa due to their accessibility and cultural familiarity [20, 21].

This research corroborates existing literature by showing a marked rise in the consumption of traditional herbs during the pandemic, consistent with studies on herbal medicine's role in boosting immunity and mitigating respiratory illnesses. Ingredients like ginger, turmeric, and lime, commonly used in Indonesian “jamu,” have well-documented antioxidant and immunomodulatory effects [22, 23]. Similar findings in other studies reinforce the efficacy of bioactive compounds in traditional medicine for managing viral infections [24].

The focus on students and young adults, a group often overlooked in traditional medicine research, adds novelty to the demographic profiling of herbal users. Moreover, the study's evidence of no significant gender or regional influence on herbal medicine consumption challenges assumptions in related studies, providing a nuanced understanding of its cultural and sociodemographic dynamics.

This research underscores the potential of integrating traditional remedies into public health strategies for pandemic preparedness and supports the ongoing exploration of herbs as antiviral agents. Its findings complement and extend prior research while opening avenues for further investigation into the modernization of traditional medicine practices.

The study participants were selected based on their gender, age, level of knowledge, domicile, and occupation. Furthermore, most were females, teenagers or young adults, undergraduates, students, and domiciled in Java. Among the 461 people selected, the majority were young adults aged 17–25.

Women often use herbal medicine for their daily needs, such as preparing additional food and supplements at home, especially during the pandemic, similar to the results [25]. This finding is inconsistent with Medisa et al. and Mulyani et al., where men were the dominant consumers [25, 26]. Based on the results, a *p* value of 0.437 > 0.05 was obtained, indicating no significant relationship between gender and herbal medicine consumption.

Regarding age, teenagers and young adults were the dominant age group. Furthermore, the result showed a significant relationship between age and the use of traditional herbs, with a *p* value of 0.006. The 17–35-year-old group comprises college students and young workers who are very mobile and concerned about COVID-19 status. They also have easy access to information from social media, which can increase their knowledge and curiosity about the interventions. This finding aligns with a previous study that found that adolescents and young adults know about herbs [26].

Based on the level of education, undergraduates (40.1) were the dominant consumers of herbs. Furthermore, there is a significant relationship between education level and consumption of herbal medicine. Undergraduates have sufficient knowledge about the high morbidity and mortality during this COVID-19 pandemic [27]. Also, we understand that using various herbal medicines is a better and more appropriate method of preventing the disease, especially when no standard treatment guide exists, and studies are still being developed.

A *p* value of 0.700 > 0.05 was obtained for the domicile factor, indicating no significant relationship with herbal medicine consumption. Compared to other islands, such as Sumatra, Kalimantan, Sulawesi, and Papua, people living on Java Island have a strong culture of using traditional ancestral herbs. Conventional medicine is often used to prevent and treat diseases (phytotherapy) [26]. Many people on the island still act as strong custodians of traditions. They also have Javanese manuscripts, such as handwritten letters from their ancestors on traditional medicine [28]. Although cultural traditions exist in other areas, they are not as strong as those in Java.

The results indicated that students with a score of 26.2 were the predominant consumers of herbal drugs, suggesting a significant level of herbal medicine use among this group. Hence, a significant relationship exists between occupation and the consumption level with a *p* value of 0.038. In another study, the self-employed group was the common users, accounting for 30.2 participants [26]. Meanwhile, a survey in Sleman, Java, reported that homemakers use the form of therapy more than others [25]. Variations in the point of view of each community cause these different results. Some students need to care for their health to avoid illness, and this increases the consumption of herbal medicine. Entrepreneurs work to earn a living, while homemakers, as the backbone of the family, care for all members by giving them herbal supplements to prevent diseases.

The significance of age and occupation in herbal medicine consumption, as opposed to gender, education level, and domicile, can be attributed to the nature of the variables and their potential influence on health behaviour. Age reflects life stages and associated health priorities, with younger adults (17–35 years) being more health-conscious and actively seeking preventive measures like herbal remedies, especially during a pandemic. This group often has greater access to information through digital platforms, enhancing their awareness and willingness to try alternative medicine. Similarly, occupation significantly affects herbal medicine usage because students and working professionals, who constituted a significant portion of respondents, might have specific needs for maintaining health and productivity, making them more likely to adopt herbal medicines.

In contrast, gender did not show significance, likely because both men and women may consume herbal medicine for similar reasons, such as improving immunity, regardless of differences in daily responsibilities. Education level, while generally influencing health literacy, may not have been a determining factor here because even individuals with varied education levels likely share a cultural familiarity with traditional herbal practices in Indonesia. Lastly, domicile, or geographical location, was not significant because herbal medicine use is widespread across Indonesia, deeply rooted in cultural traditions, and not confined to specific regions. This cultural ubiquity may override potential differences based on location, leading to similar consumption patterns across domiciles. These results highlight that while sociodemographic factors influence herbal medicine consumption, their impact varies based on cultural and behavioural nuances, with age and occupation directly shaping access, motivation, and necessity.

The specific research gap addressed by this study lies in understanding the sociodemographic factors influencing the consumption of herbal medicines as preventive healthcare during the COVID-19 pandemic, particularly in Indonesia. While traditional herbal medicine has been widely recognized and studied globally for its therapeutic and preventive properties, limited research has explored how age, occupation, gender, education, and domicile shape its usage patterns within diverse cultural and demographic settings. This study fills the gap by focusing on Indonesia, where herbal medicine is deeply ingrained in cultural practices. Yet, its adoption patterns during a modern health crisis like COVID-19 remain underexplored.

The study contributes novel insights by identifying age and occupation as significant predictors of herbal medicine consumption, a finding that contrasts with the nonsignificant roles of gender, education level, and domicile. This differentiation clarifies how lifestyle, mobility, and access to information—factors associated with age and occupation—drive preventive healthcare behaviours. Moreover, the research emphasizes the potential of herbal medicines as accessible, culturally resonant alternatives to conventional treatments during health crises, particularly in resource-constrained settings where vaccine distribution and access to modern healthcare are limited.

By addressing this gap, the study highlights the need for targeted public health strategies that leverage traditional medicine to promote preventive care, particularly among younger and working populations. It underscores the importance of tailoring interventions to demographic characteristics to maximize their effectiveness and cultural acceptability.

Traditional medicine is a widespread practice in Indonesia. Traditional medicine is a substance obtained from plants, animals, minerals, or a combination of these substances and is typically used for treatment, illness prevention, and health maintenance. Due to the influence of various elements, including culture, history, and personal views, each region uniquely uses traditional medicine. Because of the wide variety of its local cultures, Indonesia has a rich knowledge of traditional remedies. Recently, it was discovered that even urban, educated households still favour “conventional” healthcare over biological treatment [29]. Over the past 30 years, Indonesia's market for traditional and complementary medicine (TCM) has grown significantly. A wide variety of over-the-counter (i.e., nonprescription) drugs, medicines, tonics, and novel herbal or other mixes have emerged, promising increased vigor and endurance as well as defense against hardship and misery. A new market of contemporary alternative treatments is emerging in addition to the well-established traditional medicine market. This market offers a wide range of herbal energy products and stamina treatments. Indonesian traditional medicines are divided into three kinds based on production, benefits, and efficacy: jamu/herbal, standardized, and phytopharmaceuticals [30]. Jamu is a safe herbal medicine based on empirical data from generation to generation. The type of medicine used also depends on the name of each region in Indonesia.

Jamu is a native concoction produced by people in the eastern tip of the Indonesian Archipelago in Sabang and the western end in Merauke using plants that grow in their regions. The data revealed that the most commonly used herbal simplicia are ginger and turmeric, including their rhizomes. Ginger (*Zingiber officinale*) is traditionally believed to have several beneficial properties, such as increasing endurance (immunomodulator), digestive disorder reliever, cough laxative, antinausea vomiting, pain reliever, and an antibacterial, antiviral, and antifungal agent [31–33]. Furthermore, lime (*Citrus aurantifolia*) and garlic (*Allium sativum*) contain antioxidants, which help increase endurance, relieve breathing, and treat flu, fever, cough, and cold. Lemongrass (*Cymbopogon citratus*) and Temulawak (*Curcuma zanthorrhiza*) are antioxidants for various diseases and can act as anti-inflammatory, antibacterial, and antifungal agents for digestive disorders. Habatussauda (*Nigella sativa*) and *Moringa* leaves (*Moringa oleifera*) help improve the body's immune system, overcome respiratory and digestive complaints, and have antibacterial and antiviral effects. Other plants that are also used include betel leaves (*Piper betle* L.), celery (*Apium graveolens*), guava leaves (*Psidium guajava*), sambiloto (*Andrographis paniculata*), and brotowali (*Tinospora crispa*) [32, 34–37].

The herbal form is another class of medicine that contains more than one *Simplicia* ingredient, such as turmeric acid (*Curcuma longa* + *Tamarindus indica* L.), wedang uwuh, and empon empon. Furthermore, they are produced from various rhizomes, especially ginger, turmeric, and kencur (*Kaempferia galanga* L.), which are applicable as antioxidants and endurance enhancers (immunomodulators). There are also various classes of tea, such as ginger, green (*Camellia sinensis*), and rosella (*Hibiscus sabdariffa*) teas, which are widely used by brewing with hot water to warm the body and increase endurance/stamina [38].

A drug is called an OHT when it is safe and has been preclinically tested by a scientist. Its raw materials must also be standardized as a requirement for quality. In this study, OHTs that were widely consumed include Tolak Angin, Antangin JRG, OBH Herbal, Kiranti, Sehat segar, Herbacold, Mastin, and Dehaf. The results showed that Tolak Angin, Antangin JRG, OBH Herbal, and Herbacold were the most used drugs during the pandemic because they are believed to prevent COVID-19 or treat flu, colds, cough, and fever [39–41].

Furthermore, FFs are safe and produced from natural ingredients that have been standardized and proven efficacious using preclinical and clinical trials. In this study, the widely used type was Stimuno, followed by Redacid, Tensigard, Disolf, Diabetadex, Vipalbumin, Rheumeneer, and XGra. Stimuno is an herbal medicine that can increase the body's endurance or stamina. Furthermore, COVID-19 is a viral disease. Hence, people believe additional herbal supplements to increase endurance can curtail its spread [42–44].

In this sense, it is possible to interpret the rising popularity of traditional medicine as a strategy for improving one's connection to one's Indonesian roots. Since the start of the current epidemic, Indonesian culture has shown a solid dedication to traditional Javanese medical methods, particularly the traditional herbal jamu treatment. As the COVID-19 issue worsened, a new market appeared selling “Corona jamu,” a supplement that boosts the body's defenses against viruses using turmeric, ginger, and other components [15, 45]. Several Indonesian leaders have emphasized the advantages of conventional medicine in the current situation. As long as a person's body strongly resists the illness, some even publicly asserted that COVID-19 infections could heal without treatment during the initial phase. This caused public criticism and raised concerns about whether politicians purposefully withhold vital information to calm public anxiety. These insights into modern medical practice demonstrate the prevalence of traditional medicine and the blending of various healing philosophies in urban and rural Indonesia [46].

### 4.2. Time to Use Herbal Medicine

In Indonesia, people often use herbal medicine in their daily lives, and they contain secondary metabolites that can provide benefits, such as pain and fever relief, as well as increased body resistance. They also serve as antimicrobial and antifungal agents, warm the body, and help to reduce complaints of coughs, colds, and flu syndromes. COVID-19, caused by the SARS-CoV-2 virus, has several symptoms, such as fever, cough, runny nose, shortness of breath, anosmia, and other signs that vary and differ for each patient. These complaints can limit daily activities, cause pain, and death in severe cases. Hence, people believe that prevention is better than cure. Medicine that belongs to the herbal, OHT, and FF classes can help increase the body's resistance to various diseases due to their antioxidant, immunomodulatory, and cytoprotective effects [47].

The results showed that 43.2% of the participants consumed herbs before the pandemic. Indonesian cultures are associated with traditional medicine; hence, maintaining daily health often becomes a habit and necessity. The COVID-19 pandemic in recent years has increased the rate of consumption (56.4), where the highest rate of 41.4% was obtained in 2021, while 15 % was recorded in 2019.

Recent studies have expanded on the potential of Indonesian herbal medicine, particularly jamu, as an alternative treatment or complementary therapy for managing COVID-19. For instance, a narrative review highlights the antiviral and anti-inflammatory properties of plants like *Zingiber officinale* (ginger), *Curcuma longa* (turmeric), and *Citrus aurantifolia* (lime), emphasizing their role in improving immune response and preventing viral infections [48]. Similarly, studies have shown that combinations of these bioactive compounds, including curcumin and 6-gingerol, interact with SARS-CoV-2 targets, supporting their therapeutic potential against COVID-19 through molecular docking methods [49].

Further, a community-based survey recorded the use of over 59 plant species in Java and Bali for immunity boosting and respiratory health, with *Curcuma longa* and *Zingiber officinale* emerging as the most cited plants [50]. The study underscores traditional preparation methods, such as boiling herbs for oral consumption, which align with public perceptions of their efficacy during the pandemic.

The literature suggests that herbal combinations, particularly involving *Andrographis paniculata* and *Phyllanthus niruri*, provide synergistic effects in combating COVID-19. These formulations enhance the immune response and reduce inflammation, making them suitable adjunctive therapies alongside conventional treatments [51]. These findings strengthen the argument for integrating traditional herbal medicine into modern therapeutic strategies, emphasizing cultural relevance and scientific validation.

The findings of this study underscore the growing reliance on herbal medicine, particularly during health crises like the COVID-19 pandemic. This trend reflects a broader global interest in traditional remedies as complementary approaches to health and wellness, highlighting the need for further research into their efficacy and safety. Herbal ingredients such as ginger and turmeric have been shown to possess significant antiviral and immunomodulatory properties. For instance, a study by [52] demonstrated that ginger extract inhibits viral replication, while clinical trials have indicated that turmeric can enhance immune response [53]. These findings support the traditional use of these herbs in managing health conditions and highlight their potential role in contemporary therapeutic practices.

### 4.3. Cultural and Regional Influence on Herbal Medicine Consumption

The widespread use of herbal medicine in Indonesia is deeply rooted in cultural and regional traditions, with Java as a central hub for these practices. Javanese culture has preserved the knowledge of herbal medicine, often referred to as Jamu, through generations. This traditional system uses locally sourced plants commonly cultivated in home gardens, such as turmeric, ginger, and tamarind. The prominence of Jamu in Java can be attributed to the region's historical and cultural emphasis on holistic health, where herbal remedies are integrated into daily routines for preventing and treating illnesses [54, 55].

In our analysis, we explored the cultural and regional influences on herbal medicine use among participants. While the statistical results indicated that these factors were not statistically significant in relation to herbal medicine consumption, it is essential to recognize the contextual importance of these influences. Cultural and regional factors may still play a critical role in shaping individuals' attitudes and practices regarding herbal medicine, manifesting in ways that are not easily quantifiable through traditional statistical measures. We acknowledge that the lack of statistical significance may be attributed to limitations within our study, such as insufficient sample size or metrics that do not fully capture the complexities of these influences. Therefore, we recommend that future research employ qualitative methodologies to delve deeper into how cultural beliefs and regional practices affect the consumption of herbal medicine.

Java's longstanding tradition is also supported by its role as a cultural and economic center, facilitating the availability and commercialization of herbal products. Additionally, manuscripts and ancestral knowledge in the region serve as a repository of ethnopharmacological practices, reinforcing the use of Jamu in modern health practices. In contrast, other areas like Sumatra or Papua may rely more on localized plant-based remedies due to different biodiversity and cultural practices [56].

The influence of these traditions during the COVID-19 pandemic highlights how Javanese herbal medicine, including commercial products like Tolak Angin and Antangin, became prominent choices. This reflects not only the cultural trust in Jamu but also the economic accessibility of such remedies in urban and rural settings. However, the regional variation underscores the need for a broader understanding of how cultural heritage and regional resources shape herbal medicine practices across Indonesia. Future research should explore how these cultural factors interact with modern healthcare trends to sustain the relevance of traditional herbal medicine.

### 4.4. Limitations of the Study

This study acknowledges the potential demographic bias introduced by the high proportion of younger and educated individuals in the sample. Younger respondents, mainly those aged 17–35 and individuals with higher education levels, are more likely to have greater health awareness and digital connectivity, potentially influencing their herbal medicine consumption behaviours and access to health information. To mitigate this, the study employed cluster sampling to ensure representation from diverse demographic groups across Indonesia. Stratified analyses were also conducted to isolate the effects of individual variables such as age and occupation, providing robustness to the significant findings. Nonetheless, the predominance of digitally connected and health-aware groups limits the generalizability of the results to older or less-educated populations. Future research should aim to target these underrepresented groups to achieve a more comprehensive understanding of herbal medicine consumption patterns.

In light of the findings presented in this study, it is pertinent to consider the insights from [57], which discuss the role of traditional remedies in enhancing community resilience during pandemics. This article underscores the significance of herbal medicine in public health responses, particularly in resource-limited settings. The findings from our research align with this perspective, suggesting that integrating traditional remedies into pandemic preparedness strategies could enhance health outcomes and community trust in health interventions.

## 5. Conclusion

This study highlights the significant association of age and occupation with herbal medicine consumption during the COVID-19 pandemic in Indonesia, with young adults and students emerging as the primary consumers due to their active lifestyles and greater access to health information. Although gender and domicile were not significantly associated, the cultural reliance on traditional medicine, particularly in Java, underscores its integral role in Indonesian society. The widespread use of jamu and its key ingredients, such as ginger, turmeric, and lime, demonstrates the public's trust in their antiviral, antioxidant, and immunomodulatory properties. We recommend that policymakers integrate traditional remedies into health strategies to enhance pandemic preparedness, ensuring their safe and regulated use. Furthermore, we advocate for further research into the efficacy and safety of herbal medicines across diverse populations, which will provide a robust evidence base for their incorporation into mainstream healthcare practices. We can improve health outcomes and community resilience in future health crises.

## Figures and Tables

**Table 1:** Characteristics of respondents consuming herbal medicines during the COVID-19 pandemic.

**No.**	**Sociodemographic characteristics**	**Variable**	**n** ** (%)**
1	Gender	Female	306 (66.4)
		Male	155 (33.6)

2	Age	17–25	138 (29.9)
		26–35	113 (24.5)
		36–45	93 (20.2)
		46–55	83 (18.0)
		56–65	34 (7.4)

3	Education	Elementary–high school	159 (34.6)
		Diploma	12 (2.6)
		Bachelor	185 (40.1)
		Master–doctoral	105 (22.8)

4	Domicile	Sumatra	91 (19.7)
		Java	289 (62.7)
		Kalimantan Bali NTB	40 (8.6)
		Sulawesi Papua	41 (8.9)

5	Occupation	Housewife	55 (11.9)
		Worker	103 (22.4)
		Self-employed	104 (22.6)
		Student/student	121 (26.2)
		Others	78 (16,9)

*Note:* Number of respondents = 461 people.

**Table 2:** Herbal medicine consumption in Indonesia during the pandemic.

**Herbal class medicine**	**N**	**(%)**
Herbal/Jamu	289	62.7
OHT (standardized herbal medicine)	107	23.2
FF (phytopharmaceuticals)	65	14.1
Total	461	100.0

**Table 3:** Herbal medicine consumption during the COVID-19 pandemic in Indonesia.

**Herbal class medicine**	**n**	**Percentage (%)**
Herbal/Jamu		
Ginger/red ginger (*Z. officinale*/*Z. officinale var. rubrum*)	693	18.70
Turmeric (*Curcuma longa* Linn)	375	10.12
Lime (*Citrus aurantifolia*)	316	8.53
Kunyit asem (*Curcuma longa* + *Tamarindus indica* L.)	242	6.53
Garlic (*Allium sativum*)	218	5.88
Lemongrass (*Cymbopogon citratus*)	213	5.75
Ginger tea	213	5.75
Green tea (*Camellia sinensis*)	190	5.13
Curcuma (*Curcuma zanthorrhiza*)	183	4.94
Habatusaudah (*Nigella sativa*)	168	4.53
Wedang uwuh	163	4.40
Empon empon	119	3.21
*Moringa* leaves (*Moringa oleifera*)	114	3.08
Betel leaves (*Piper betle* L.)	104	2.81
Celery (*Apium graveolens*)	76	2.05
VCO	76	2.05
Rosella tea (*Hibiscus sabdariffa*)	55	1.48
Guava leaves (*Psidium guajava*)	53	1.43
Sambiloto (*Andrographis paniculata*)	50	1.35
Cajuput candy (*Melaleuca cajuputi*)	29	0.78
Make your herbal concoction	27	0.73
Brotowali (*Tinospora crispa*)	23	0.62
Beras kencur (*Oryza sativa* L. + *Kaempferia galanga* L.)	5	0.13
Total	3705	100
Standardized herbal medicine (OHT)		
Tolak angin	345	45.69
Antangin JRG	144	19.07
OBH Herbal	136	18.01
Kiranti	61	8.08
Sehat segar	29	3.84
Others (Herbacold, Mastin, Dehaf)	40	5.29
Total	755	100
Phytopharmaceuticals (FF)		
Stimuno	413	77.48
Redacid	61	11.44
Tensigard	15	2.81
Dissolve	12	2.25
Diabetadex	11	2.06
Vipalbumin	9	1.69
Rheumeneer	8	1.5
X Gra	4	0.75
Total	533	100

**Table 4:** Herbal medicine consumption during the COVID-19 pandemic in Indonesia.

**Herbal medicine consumption**	**N**	**Percentage (%)**
During the COVID-19 pandemic	191	41.4
During the COVID-19 pandemic (last year)	71	15
Before the COVID-19 pandemic (last 2–3 years)	199	43.2
Total	461	100

**Table 5:** The relationship between sociodemographic factors and herbal medicine consumption in Indonesia during the COVID-19 pandemic.

**Sociodemographic characteristics**	**Herbal class**			**Total**	**p** ** value**
	**Herbal**	**OHT**	**FF**		
Gender					
Female	198	68	40	306 (66.4)	0.437
Male	91	39	25	155 (33.6)	
Age					
17–25	79	30	29	138 (29.9)	0.006
26–35	77	21	15	113 (24.5)	
36–45	61	25	7	93 (20.2)	
46–55	44	28	11	83 (18.0)	
56–65	28	3	3	34 (7.4)	
Education					
Elementary–high school	94	35	30	159 (34.6)	0.182
Diploma	11	1	0	12 (2.6)	
Bachelor's degree	116	45	24	185 (40.1)	
Master's and doctorate	68	26	11	105 (22.8)	
Domicile					
Sumatra	56	22	13	91 (19.7)	0.700
Java	186	64	39	289 (62.7)	
Kalimantan Bali NTB	24	12	4	40 (8.6)	
Sulawesi Papua	23	9	9	41 (8.9)	
Occupation					
Housewife	37	11	7	55 (11.9)	0.038
Worker	66	29	8	103 (22.4)	
Self-employed	59	28	17	104 (22.6)	
Student	68	28	25	121 (26.2)	
Others	59	11	8	78 (16,9)	

*Note:* Number of respondents = 461 people.

## Data Availability

All data generated or analyzed during this study are available within the paper.
